# Metabolic change in monocytes and postoperative morbidity after major abdominal surgery in elderly patients: A prospective cohort study

**DOI:** 10.1016/j.heliyon.2024.e28137

**Published:** 2024-03-22

**Authors:** Yi Zhao, Mengchan Ou, Xuechao Hao, Tao Zhu

**Affiliations:** Department of Anesthesiology, and the Research Units of West China (2018RU012) - Chinese Academy of Medical Sciences, West China Hospital of Sichuan University, Sichuan University, Chengdu, China

**Keywords:** Abdominal surgery, Elderly patients, Postoperative complications, Metabolic change, Risk assessment

## Abstract

**Background:**

Postoperative complications in aging patients remain a significant cause of increased costs, hospital length of stay, and patient distress. Although alterations in energy metabolism have been closely linked to aging process and surgery, it is still unclear whether metabolic changes during surgery is associated with postoperative complications in elderly patients. This study was conducted to investigate whether metabolic changes during surgery predicts postoperative complications in elderly patients.

**Methods:**

We conducted a prospective single-center observational cohort study. 244 adults (aged ≥65 years) who were scheduled for elective major non-cardiac surgery were recruited. Blood samples for each patient were taken before and after surgery. All patients were randomly divided into two groups (122 in each group), then oxygen consumption rate (OCR) or extracellular acidification rate (ECAR) was measured on isolated monocytes in each group.

**Results:**

14 of 110 (12.7%) patients went through OCR measurement and 15 of 122 patients (12.3%) went through ECAR measurement experienced moderate to severe complications. Overall, there was an intensification of glycolysis in monocytes after surgery. Among all variables, only the change (preoperative -postoperative) of glycolytic reserve (GR)/glycolysis (G) and GR/non-glycolytic acidification (NG) were predictors of moderate to severe complications (AUC = 0.70; 95% CI, 0.56–0.81; *P* = 0.019 and AUC = 0.67; 95% CI, 0.55–0.80; *P* = 0.031). Decreased postoperative GR/G were associated with worse postoperative complications (RR = 9.08; 95% CI, 1.23–66.81; *P* = 0.024).

**Conclusions:**

Compared with mitochondria function, the change of glycolytic function in monocyte was more valuable in predicting postoperative complications after major abdominal surgery. Our study gave us a new insight into identifying patients at high risk in aging patients.

## Highlights

• This is the first clinical study investigated mitochondrial respiration and glycolytic function of peripheral monocytes at the same time.
• This study was conducted to investigate whether metabolic changes during surgery could predict postoperative complications in elderly patients.
• Compared with mitochondria function, the change of glycolytic function in monocyte was more valuable in predicting postoperative complications after major abdominal surgery.

## Abbreviations

GCRI: Goldman Cardiac Risk Index
BNP: B-type natriuretic peptide
NGAL: neutrophil gelatinase-associated lipocalin
BHI: Bioenergetic Health Index
OCR: oxygen consumption rate
ECAR: extracellular acidification rate
G: glycolysis
GC: glycolytic capacity
GR: glycolytic reserve
NG: non-glycolytic acidification
CDC: Clavien-Dindo Classification
AUC: area under the curve
ROC: receiver operating characteristic
FDR: False Discovery Rate
CI: confidence interval
BR: basal respiration
ATP-P: ATP production
PL: proton leak
MR: maximal respiration
NMR: non-mitochondrial respiration
SRC: spare respiration capacity
RR: relative risk

## 1 Introduction

In recent decades, dying within 30 days after surgery has declined [1]. However, this risk is still excessively high for aging patients [2]. Postoperative complications in aging patients remain a significant cause of increased costs, hospital length of stay, and patient distress [3]. Identifying patients who are most at high risk before surgery allows early interventions and improves the overall outcomes, in particular by focused on the ages and pre-existing comorbidities of the surgical cases [4,5]. Traditionally, surgical risk-stratification are guided by the Goldman Cardiac Risk Index (GCRI), the American Society of Anesthesiologists Physical Status Classification System, Acute Physiology and Chronic Health Evaluation-II, and more [6]. However, these risk-stratification tools ignore the influence of surgery and anesthesia, and could not be dynamically assessed.

Researches have indicated several biomarkers for predicting perioperative complications, including B-type natriuretic peptide (BNP) [7], neutrophil gelatinase-associated lipocalin (NGAL) [8] and S100B protein [9]. These predictive biomarkers reflect heterogeneity in individual responses to surgery and could not be dynamically assessed perioperatively. However, as damage markers of organs, one biomarker only show predictive value in one specific organ or system. For example, as a marker of tubular damage, NGAL has been demonstrated closely associated with acute kidney injury (AKI) after cardiac and colorectal surgery [8,10]. Therefore, there is a pressing need for new biomarkers to predict the overall risk of aging people during perioperative period.

Recent study has demonstrated that changes in energy metabolism were closely associated with age-linked comorbidities [11]. Mitochondria transplantation is emerging as an effective strategy for improving cognitive deficits and heart failure in rodents [12,13], which reveals the link between mitochondrial function and prognosis of diseases. According to previous evidence, energy production progressively decreases with age in all organisms for declining mitochondrial activity [14]. Common comorbidities in aging people, including neurodegeneration [15], diabetes [16] and sarcopenia [17] have all been shown to be associated with a deterioration in mitochondria. In addition, surgical procedures and general anesthesia also exert a dramatic influence on metabolism, including trauma-associated stress responses, increased secretion of glucocorticoids and catecholamines and a reduction in body temperature [18,19]. Thus, elderly patients with pre-existing mitochondrial malfunctions may be more vulnerable to perioperative disturbances of metabolism. However, there is few published studies concerning the metabolic changes after surgery in aging patients.

The blood-cell based measures of metabolism provides a minimally invasive method to evaluate systemic metabolism for diagnostic purposes [20]. More recently, Chacko et al. proposed a Bioenergetic Health Index (BHI) to measure a patient's composite mitochondrial profile, using monocytes isolated from a blood sample [21]. Indeed, one previous study found that the BHI was markedly reduced in monocytes isolated from postoperative pericardial fluid and the blood taken from patients who had underwent heart surgery [22]. These results suggested the peripheral monocytes are suitable for measurements of systemic metabolism. Unfortunately, the sample size of previous studies on the BHI were very small, and thus failed to illustrate the relationship between systemic metabolism and surgical outcomes.

In summary, the study was conducted to investigate: (1) metabolic changes during surgery by testing mitochondrial respiration and glycolytic function of peripheral monocytes; (2) if the metabolic change could predict complications after major abdominal surgery in elderly patients. As metabolism is emerging as the therapy target for the treatment of many diseases like diabetes [23], heart failure [12] and cognitive impairment [24], our study might give a new insight into identifying patients at high risk for surgery and improving surgical outcome.

## 2 Methods

### 2.1 Patients

This is a prospective single-center observational cohort study. Ethic approval for this study has been obtained from the Ethics Committee on Biomedical Research, West China Hospital of Sichuan University, Sichuan, China (No. 2019-624). This trial was registered at Chinese Clinical Trial Registry (ChiCTR1900026223). Consent was provided by all participating patients or their guardians. Patients underwent major abdominal surgery (>3h) under general anesthesia between Oct 2019 and Dec 2021were included. The inclusion criteria were: patients who underwent major abdominal surgery (>3 h) between Oct 2019 and Dec 2021; aged ≥65 years; male or female; ASA physical status I–III; patients mentally competent enough to provide informed written consent. Exclusion criteria were: previous allergic reaction to general anesthetics; hematological disease; psychiatric disorders; learning difficulties; hemodynamic instability; or had received a previous organ transplant. As it takes 8 h for monocyte isolation and metabolic flux analysis, only patients given general anesthesia before 9:00 a.m. were included. Considering the monocytes isolated from 10 ml blood were just enough for the measurement of oxygen consumption rate (OCR) or extracellular acidification rate (ECAR), the patients were randomly assigned into two groups using random number table.

### 2.2 Metabolic function tests

***Blood collection:*** Venous blood samples (10 mL) were collected in EDTA-containing tubes, immediately preserved at 4 °C and sent to laboratory within 30 min. Preoperative blood samples were collected from patients whose anesthesia began before 9:00 a.m., for it took about 8 h for monocyte isolation and metabolic flux analysis. Postoperative blood samples were taken at 8:00–9:00 a.m., the first day after surgery.

***Monocyte isolation:*** Firstly, the blood was centrifuge at 500 g for 10 min at room temperature. Then, the leucocyte-enriched layer on top of the red blood cell pellet was collected and diluted (1:4) with RPMI medium (Seahorse Bioscience, Agilent, USA). The diluted RPMI was applied to a Histopaque**®** density gradient (Sigma-Aldrich, Merck, USA) at room temperature and centrifuged without brake at 700g for 30 min. Finally, the peripheral blood mononuclear cells (PBMCs) were harvested. Then CD14^+^ monocytes were isolated from PBMCs with magnetic cell sorting (MACS) technique (Milteneyi Biotec) using superparamagnetic iron-dextran microbead-labeled anti-CD14 antibodies according to manufacturer's instructions. PBMC were incubated with the labeled anti-CD14 antibodies at 4 1C for 15 min before applying the cells to the column placed in the magnetic field. Trypan blue will be added into the cell samples to get the viability (>90 %) and density of living cells.

**Fig. 1:**
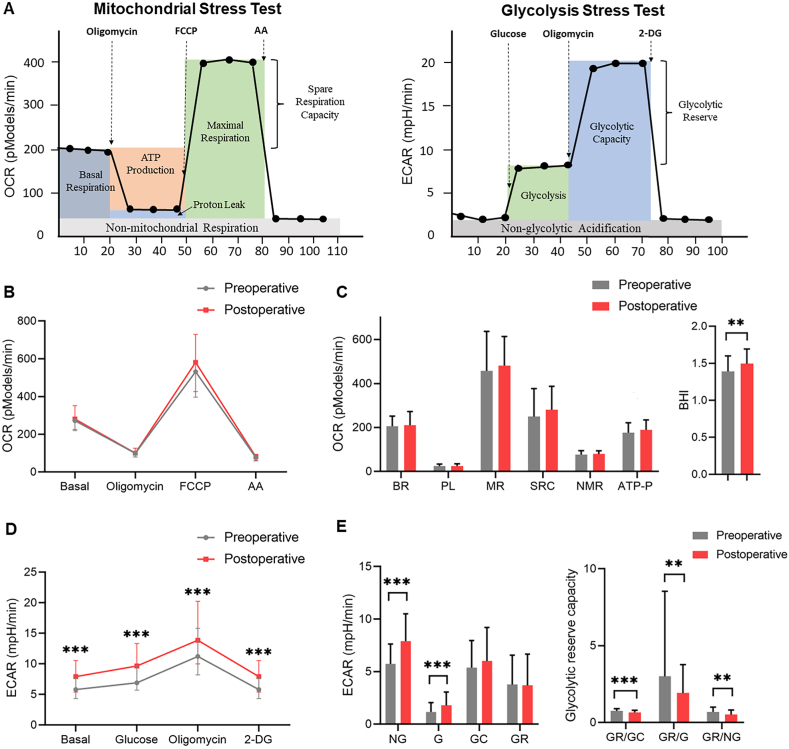
Metabolic changes during the perioperative period. (A) Example of mitochondrial stress test and glycolysis stress test assay showing calculated parameters. (B–C) Pre and postoperative OCR changes. (D–E) Pre and postoperative ECAR changes. AA: antimycin A; 2-DG: 2-deoxyglucose; OCR, oxygen consumption rate; ECAR, extracellular acidification rate; BR, basal respiration; PL, proton leak; MR, maximal respiration; SRC, spare respiration capacity; NMR, non-mitochondrial respiration; ATP-P, ATP production; BHI, bioenergetic health index, NG, non-glycolytic acidification; G, glycolysis; GC, glycolytic capacity; GR, Glycolytic Reserve.

***Metabolic flux analysis*:** The real-time measurements of OCR or ECAR were measured using Seahorse XF8 analyzer (Seahorse Bioscience, Agilent, USA). Based on density calculated before, diluted monocytes (about 300,000 living cells in 200 μl in each well) were plated onto rat tail collagen I (Corning, USA) coated 8-well assay plates. The cells were attached to the bottom of the plate by centrifugation at 40*g* without brake, and allowed 30 min attachment time at 37 °C in a non-CO_2_ incubator. Then real-time measurements of the OCR and ECAR were performed [25]. OCR was assessed using a protocol for a mitochondrial stress test. FCCP (0.6 mM), oligomycin (0.5 mg/mL) and antimycin A (10 mM) were injected in sequence to assess respiratory parameters, which included basal respiration (BR), ATP production (ATP-P), proton leak (PL), maximal respiration (MR), non-mitochondrial respiration (NMR). Spare respiration capacity (SRC) was calculated by the subtraction of the BR from MR. Optimum inhibitor and activator concentrations were used as previously described [26,27]. BHI = log [ (reserve capacity)^a^ × (ATP-linked)^b^/(non-mitochondrial)^c^ × (proton leak)^d^ ] [28]. ECAR was assessed using a glycolysis stress test with sequential injections of oligomycin (1.0 μg/mL), glucose (5 mM) and 2-deoxyglucose (100 mM). Parameters calculated from the glycolysis stress tests include glycolysis (G), the glycolytic capacity (GC), glycolytic reserve (GR), and non-glycolytic acidification (NG). GR/GC, GR/G and GR/NG ratio were calculated to reflect glycolytic reserve capacity. A visualization of parameter calculations is shown in Fig. 1 A.

### 2.3 Anesthesia and surgical procedures

Each patient received standard assessments before surgery, including medical history, physical examination, a 12-lead electrocardiogram and laboratory blood tests. Additional examinations were carried out if deemed necessary. Existing preoperative comorbidities were determined based on medical histories and patient self-reporting. They included hypertension, coronary artery disease, myocardial infarction, positive exercise, nuclear or electrocardiogram stress tests, diabetes mellitus, arrhythmias, smoker; chronic obstructive pulmonary disease (COPD) and cerebrovascular disease (specific definitions were listed in Supplemental method).

All patients were monitored with pulse oximetry, electrocardiography, noninvasive arterial blood pressure measurements and the BIS score (BIS, Covidien LLC, USA). General anesthesia was induced with intravenous injection of propofol (1.5–2.5 mg/kg) and sufentanil (0.4 μg/kg), then cisatracurium (0.2 mg/kg) or vecuronium (0.1 mg/kg) were also then given. After intubation, patients were ventilated with a fresh gas flow of 2L/min of oxygen and air. Anesthesia was maintained with an intravenous remifentanil infusion and sevoflurane or desflurane inhalation, or propofol TCI. At skin closure, neostigmine (20 μg/kg) and atropine (10 μg/kg) were given to reverse residual neuromuscular block unless contraindicated. After the surgery, patients were transferred to the post-anesthesia care unit (PACU), ward or intensive care unit (ICU) according to different statues.

### 2.4 Postoperative follow-up

Patients were monitored each day throughout their stay in hospital after surgeries. After discharge from hospital, each patient was contacted 30 days after surgery. The degree of any complications was categorized using a modified Clavien-Dindo Classification (CDC) scheme [29]. The primary outcome in the present study was moderate to severe complications (≥CDC grade 3).

### 2.5 Analysis of data

Statistical analysis was performed using SPSS software (version 23, IBM, USA) and GraphPad Prism (version 9, GraphPad Software, USA). For normally distributed variable, data was expressed as mean ± standard deviation (SD); for non-normally distributed variable, data were reported as medians and inter-quartile range (IQR). All statistical tests were 2 sided and significance was assumed at *P<*0.05. Comparisons of parameters before and after the surgery were performed by the Wilcoxon match-pairs signed rank test, other comparisons were performed by the Mann-Whitney test. The changes of parameters were calculated as preoperative minus postoperative. ROC analysis was conducted to evaluate potential predictive factors for postoperative complications. Based on the changes of parameters (preoperative -postoperative) was greater than zero or not, the relative risk (RR) of postoperative complications was estimated using a chi-squared test. The AUC and RR were expressed as median and 95% confidence interval (CI).

Sample size calculation was performed by MedCalc software (version 19, Reachsoft, China), by caculating the area under the curve (AUC) of the receiver operating characteristic (ROC) curves. Under the assumption of outcome event rate of 9–12% (based on pre-experiment), a moderately good AUC of 0.8, a sample size of 110 patients had 90% power to detect this clinically relevant difference in AUC values (2-sided α 0.05). To account for the 10% of patients potentially lost to follow-up, we recruited of 244 patients (122 in each group). When the study was completed, missing data were excluded from the analyses.

## 3 Results

**Fig. 2:**
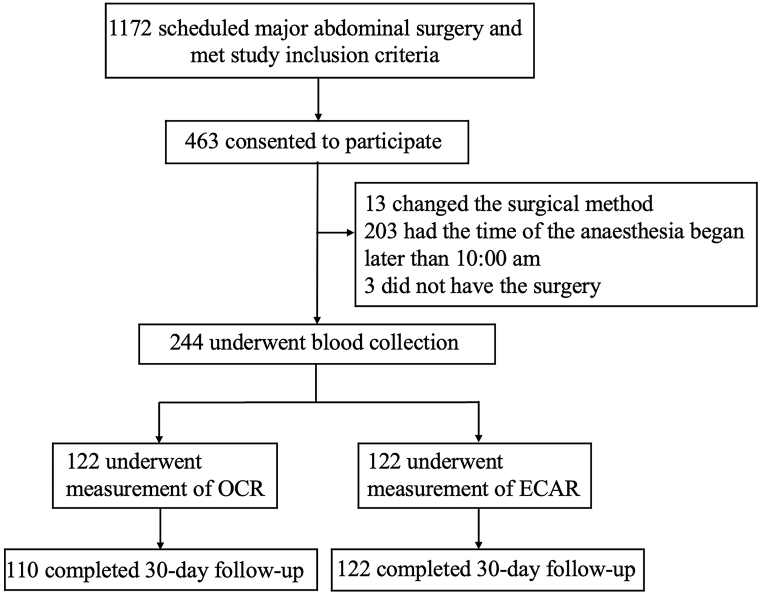
**Flowchart showing the participant recruitment and follow-up.** OCR: oxygen consumption rate; ECAR: extracellular acidification rate.

From October 2019 to December 2021, 1172 patients were eligible for inclusion in the study, and 463 consented to participate (Fig. 2). Of these, 203 were excluded due to changes in the surgical method, anesthesia began later than 09:00 a.m. or cancellation of surgery. A total of 244 were enrolled: 122 in OCR group and 122 in ECAR group. Finally, 110 patients in OCR group and 122 patients in ECAR group finished the 30 days follow-up. Baseline characteristics of participants were reported in Table 1. In both cohorts of subjects, groups shown no difference significantly on demographic or anthropometric measures, preoperative comorbidities or procedure types.

**Table 1:** Participant baseline characteristics and type of surgery.

Characteristics	Value, mean ± SD/median(IQR)/n (%)		
	OCR (110)	ECAR (122)	*P* value
Demographic characteristics			
Age (years)	70.00 [66.75, 75.00]	70.00 [67.00, 74.00]	0.715
Female sex	35 (31.81%)	42 (34.42%)	0.674
Height (cm)	162.06 ± 7.95	163.04 ± 8.32	0.363
Weight (kg)	59.74 ± 10.08	60.27 ± 9.36	0.679
**ASA - PS**			
Class 2	68 (61.82%)	77 (63.11%)	0.536
Class 3	42 (38.18%)	45 (36.89%)	0.893
**Motion equivalent**			
>6 MET	25 (22.73%)	32 (26.23%)	0.536
3–6 MET	78 (70.91%)	83 (68.03%)	0.635
<3 MET	7 (6.36%)	7 (5.74%)	0.842
**Comorbidities**			
Hypertension	30 (27.27%)	32 (26.22%)	0.858
Coronary artery disease	5 (4.54%)	6 (4.92%)	0.894
Arrhythmias	7 (6.36%)	6 (4.92%)	0.633
Diabetes mellitus	16 (14.54%)	17 (13.93%)	0.894
Current or recent smoker	26 (23.63%)	30 (24.59%)	0.865
COPD	8 (7.27%)	9 (7.37%)	0.976
Cerebrovascular disease	2 (1.81%)	2 (1.63%)	0.917
**Surgery**			
Open surgery	65 (59.10%)	75 (61.48%)	0.618
**Procedure type**			
Hepatobiliary	24(21.81%)	28 (22.95%)	0.836
Gastrointestinal	34(30.90%)	38 (31.14%)	0.969
Pancreatic	13(11.81%)	14 (11.48%)	0.935
Urological	39(35.45%)	44 (36.06%)	0.923

IQR, inter-quartile range; OCR, oxygen consumption rate; ECAR, extracellular acidification rate; COPD, Chronic obstructive pulmonary disease; ASA-PS, American Society of Anesthesiologists Physical Status; MET, Metabolic equivalent.

Metabolic changes during the perioperative period were shown in Supplemental Table 1 and Fig. 1 B-E. In OCR measurement, only BHI increased from 1.39 (IQR: 1.11, 1.60) to 1.49 (IQR:1.14, 1.69) after surgery (*P* = 0.008). In ECAR group, NG increased from 5.74 (IQR: 4.26, 7.62) to 7.91 (IQR: 5.40, 10.50) after the surgery (*P* < 0.001). and G increased from 1.17 (IQR: 0.61, 2.01) to 1.78 (IQR: 1.05, 3.05) after the surgery (*P* < 0.001). But GR/GC, GR/G and GR/NG ratio decreased from (0.75, IQR: 0.61, 0.90; 2.94, IQR: 1.50, 8.54 and 0.69, IQR: 0.41, 0.10) to (0.66, IQR: 0.54, 0.79; 1.92, IQR: 1.16, 3.77 and 0.51, IQR: 0.33) after surgery (*P* < 0.001, *P* = 0.001 and *P* = 0.002).

Totally, 14 of 110 (12.7%) patients underwent OCR and 15 of 122 (12.3%) patients (12.3%) underwent ECAR measurement experienced moderate or severe complications within 30 days after surgery. Comparations of paraments between complication positive and complication negative patients were shown in Supplemental Table 2. In OCR measurement, there was no difference between complication positive and negative patients. In ECAR measurement, postoperative GR/NG was higher for patients with postoperative complications ≥ CDC grade 3 (0.53, IQR: 0.34, 0.81) than those without complications (0.35, IQR: 0.15, 0.57; *P* = 0.047). The change of GR/G and GR/NG (preoperative - postoperative) showed significant difference (*P* = 0.018 and *P* = 0.029) in complication positive group (3.01, IQR: 0.57, 8.73 and 0.32, IQR: 0.12, 0.63) and complication negative group (0.52, IQR: −1.11, 2.99 and 0.30, IQR: −0.15, 0.47).

**Fig. 3:**
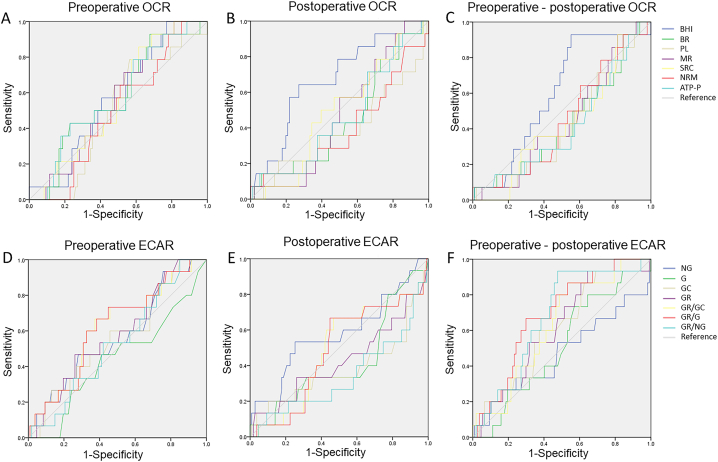
**Results of ROC analysis for all paraments in predicting complications within 30 days after surgery.** OCR, oxygen consumption rate; ECAR, extracellular acidification rate; BR, basal respiration; PL, proton leak; MR, maximal respiration; SRC, spare respiration capacity; NMR, non-mitochondrial respiration; ATP-P, ATP production; BHI, bioenergetic health index, NG, non-glycolytic acidification; G, glycolysis; GC, glycolytic capacity; GR, Glycolytic Reserve.

The ROC analysis revealed the important predictive values of all paraments. As shown in Table 2 and Fig. 3, OCR had no predictive effect on postoperative complications. In ECAR measurement, postoperative GR/NG could predict postoperative complications ≥ CDC grade 3 (AUC = 0.66; 95% CI, 0.50–0.82; *P* = 0.046). The AUC of the changes of GR/G and GR/NG ratio (preoperative - postoperative) were 0.69 (95% CI, 0.56–0.81; *P* = 0.019) and 0.67 (95% CI, 0.55–0.80; *P* = 0.031) respectively.

**Table 2:** Predictive performance of diﬀerent paraments of mitochondrial function for 30-day complications ≥ CDC grade 3.

Variables	OCR group		Variables	ECAR group	
	AUC (95%CI)	*P* value		AUC (95%CI)	*P* value
Preoperative					
BR	0.57 (0.43, 0.72)	0.372	NG	0.57 (0.41, 0.72)	0.398
PL	0.50 (0.36, 0.63)	0.954	G	0.44 (0.28, 0.60)	0.459
MR	0.56 (0.42, 0.69)	0.490	GC	0.56 (0.40, 0.71)	0.440
SRC	0.56 (0.42, 0.69)	0.490	GR	0.58 (0.43, 0.73)	0.315
NMR	0.50 (0.36, 0.63)	0.957	GR/GC	0.62 (0.47, 0.76)	0.150
ATP-P	0.58 (0.43, 0.73)	0.346	GR/G	0.62 (0.47, 0.76)	0.139
BHI	0.57 (0.43, 0.70)	0.407	GR/NG	0.53 (0.39, 0.67)	0.726
Postoperative					
BR	0.55 (0.39, 0.70)	0.587	NG	0.58 (0.40, 0.72)	0.334
PL	0.61 (0.43, 0.78)	0.208	G	0.58 (0.41, 0.74)	0.334
MR	0.52 (0.39, 0.65)	0.809	GC	0.62 (0.44, 0.80)	0.139
SRC	0.50 (0.36, 0.65)	0.982	GR	0.57 (0.40, 0.75)	0.356
NMR	0.59 (0.42, 0.75)	0.288	GR/GC	0.51 (0.36, 0.67)	0.867
ATP-P	0.53 (0.38, 0.69)	0.687	GR/G	0.52 (0.36, 0.67)	0.845
BHI	0.66 (0.51, 0.81)	0.054	GR/NG	0.66 (0.50, 0.82)	0.046
Preoperative-postoperative					
BR	0.57 (0.41, 0.73)	0.404	NG	0.53 (0.35, 0.71)	0.726
PL	0.58 (0.44, 0.73)	0.322	G	0.51 (0.37, 0.66)	0.867
MR	0.55 (0.41, 0.70)	0.527	GC	0.60 (0.46, 0.75)	0.196
SRC	0.57 (0.42, 0.71)	0.427	GR	0.61 (0.47, 0.75)	0.168
NMR	0.55 (0.40, 0.70)	0.557	GR/GC	0.63 (0.50, 0.76)	0.109
ATP-P	0.58 (0.42, 0.74)	0.362	GR/G	0.70 (0.56, 0.81)	0.019
BHI	0.60 (0.46, 0.74)	0.228	GR/NG	0.67 (0.55, 0.80)	0.031

OCR, oxygen consumption rate; ECAR, extracellular acidification rate; CDC, Clavien-Dindo Classification; AUC, area under the curve; BR, basal respiration; PL, proton leak; MR, maximal respiration; SRC, spare respiration capacity; NMR, non-mitochondrial respiration; ATP-P, ATP production; BHI, bioenergetic health index, NG, non-glycolytic acidification; G, glycolysis; GC, glycolytic capacity; GR, Glycolytic Reserve.

RR values were calculated to identify the contribution of perioperative metabolic changes to the risk of postoperative complication (Table 3). In OCR measurement, decreased BR after surgery (preoperative – postoperative >0) was associated with moderate to severe postoperative complications (RR = 3.22; 95% CI, 1.01–8.99; *P* = 0.037), while increased BHI was associated with postoperative complications (RR = 0.23; 95% CI, 0.05–0.99; *P* = 0.025). In ECAR measurement, decreased postoperative GR/G were associated with worse postoperative complications (*P* = 0.032 and RR = 9.08; 95% CI, 1.23–66.81; *P* = 0.024).

**Table 3:** Relative risk of moderate to severe postoperative complications in OCR and ECAR group.

Variables	OCR group		Variables	ECAR group	
	Relative risk (95%CI)	*P* value		Relative risk (95%CI)	*P* value
Preoperative-postoperative					
BR	3.00 (1.01, 8.99)	0.037	NG	1.19 (0.44, 3.25)	0.964
PL	1.67 (0.60, 4.68)	0.318	G	0.15 (0.02, 1.115)	0.051
MR	2.09 (0.72, 5.61)	0.172	GC	1.89 (0.72, 4.98)	0.190
SRC	1.77 (0.64, 4.63)	0.275	GR	1.35 (0.52, 3.48)	0.537
NMR	1.20 (0.45, 3.19)	0.715	GR/GC	4.80 (0.90, 16.07)	0.044
ATP-P	2.16 (0.77, 6.03)	0.130	GR/G	4.07 (0.96, 17.25)	0.032
BHI	0.23 (0.05, 0.99)	0.025	GR/NG	9.08 (1.23, 6.81)	0.024

OCR, oxygen consumption rate; ECAR, extracellular acidification rate; BR, basal respiration; PL, proton leak; MR, maximal respiration; SRC, spare respiration capacity; NMR, non-mitochondrial respiration; ATP-P, ATP production; BHI, bioenergetic health index, NG, non-glycolytic acidification; G, glycolysis; GC, glycolytic capacity; GR, Glycolytic Reserve.

## 4 Discussion

The first result reported is metabolic changes during the perioperative period in aging people. In OCR group, our results indicated an increase BHI after surgery. However, in another study carried by Philip and colleagues, there was a significant decrease in BHI value of monocytes in postoperative patients when compared with healthy people [22]. Different trial design may account for this difference: we compared blood samples of the same patients, but Philip and colleagues compared results between postoperative and healthy groups. What's more, there was an elevation or decrease of postoperative BHI in different individuals in this study, while BHI of all patients after cardiac surgery were lower than health group in study of Philip et al. [22]. Different types of surgery might account for this discrepancy in BHI result. Cardiac surgery and cardiopulmonary bypass exerted far more serious oxidative stress and inflammation than major abdominal surgery, which might cause extreme inhibition of mitochondrial function.

In RCAR group, NG, G and GC increased after the surgery, while GR/GC, GR/G and GR/NG ratio decreased. Our results indicated that surgery imposed greater influence on glycolytic function when compared with mitochondrial respiration. Increased NG, G and GC showed increased glycolytic function in monocytes after surgery. In this study, GR/GC, GR/G and GR/NG ratio were calculated to represent glycolytic reserve capacity. Decreased GR/GC, GR/G and GR/NG ratio indicated a depressed glycolytic reserve capacity in aging patients after surgery. Some authors believe a shift from mitochondrial respiration to glycolysis is an important protective-adaptive mechanism [30]. Indeed, aerobic glycolysis provides energy relatively quickly, which is beneficial for intense immune response [31]. This transition to glycolysis was first found by the biochemist Otto Warburg in cancer cells [32], but is now known to be a rare phenomenon in various disease, like neurodegenerative diseases [33] and species [34]. Interestingly, our study showed the Warburg effect also appeared during surgery, which supported the believe that the intensification of glycolysis adaptive effect in acute phase [30]. However, a lower glycolytic activity after surgery was shown in lymphocytes in another study; also, the anticipated increase in glycolytic activity following inhibition of mitochondrial respiration was reduced when compared with preoperative values [35]. This difference may result from different bioenergetic profiles in the monocytes and lymphocytes [36] and different protocols of metabolic flux analysis.

The primary result in this study was the relationship between perioperative metabolic change and postoperative complications. Our result suggested glycolytic function of monocytes was more valuable in predicting postoperative complications after major abdominal surgery in aging population, and the change of GR/NG ratio was most relevant to postoperative outcomes. The results of ROC curve analysis demonstrated that all preoperative and postoperative paraments of OCR had no predictive effect on postoperative complications ≥ CDC grade 3. Although previous studies suggested that BHI may act as a biomarker for oxidative stress in patients with metabolic disorders [22,28], BHI showed no predictive value in the study. For one reason, this may represent the ideal BHI equation to identify patients most likely for develop postoperative complications. For another, previous study reported that aging impairs mitochondrial respiration in classical monocytes by reducing MR and SRC [37]. The decline in mitochondrial function with age may result in reduced mitochondrial response to surgical stress. In ECAR group, postoperative GR/NG ratio, changes of GR/G ratio and GR/NG ratio had predictive values. These results indicated the glycolysis stress test were more valuable in predicting postoperative complications. Also, as GR/G and GR/NG ratio represented GR capacity, the AUC of changes of GR/G and GR/NG ratio indicated decreased glycolytic reserve capacity predicted poor outcomes after surgery. As described before, our result showed an intensification of glycolysis during surgery. It has been reported extensively that surgery increased the concentrations of catecholamines, growth hormone, glucagon and cortisol [38]. As insulin is an inhibitor of this process [39], glycemic control may affect surgical outcomes in aging patients.

We reported that the RR of BR, BHI and GR/NG ratio were statistically and clinically significant. In the study of Philip et al., patients with low BHI values in peripheral blood monocytes developed atrial fibrillation [22]. This result was consistent with our finding that the change of BHI was relevant to postoperative complications. In our study, the RR of changes of BHI was 0.223, which suggested patients with higher postoperative BHI have higher risk of postoperative complications. However, Philip's research found patients with low BHI values had a higher risk of postoperative atrial fibrillation [22]. As mentioned above, BHI equation and impaired mitochondrial respiration in elderly population may account for this result [37]. Although the results of ROC curve analysis represented limited predictive ability in ECAR group, the RR of the change of GR/NG ratio told us that the probability of postoperative complications in patients with decreased postoperative GR/NG ratio was nine times of those with increased GR/NG ratio after surgery.

One limitation of the present study was the relatively small sample size. However, up to now, our study is largest clinical trial that has investigated the mitochondrial functions of blood cells. Secondly, patients lost to follow-up appeared only in OCR group, withdraw bias was imbalance between two groups. Thirdly, we did not include intraoperative characteristics like operation time, blood loss or blood transfusion in this study. Fourthly, the time needed for monocyte isolation and metabolic flux analysis was too long to become an efficient clinical test.

In conclusion, this is the first clinical study investigated mitochondrial respiration and glycolytic function of peripheral monocytes at the same time. Unexpectedly, it seemed that glycolysis played a greater role in metabolic reprogramming following surgery. And the change of glycolytic function was more valuable in predicting postoperative complications after major abdominal surgery in aging population. Although mitochondrial function measurements have often been proposed to have potential diagnostic and prognostic uses, our results failed to demonstrate it as a valuable predictor of various types of postoperative complications. The results indicated that a GR capacity may be more important in an aging population for protecting responses to surgical stress. Recently, regulating metabolism has become a potential therapy in multiple disease states, including drug, exercise training, nutrition support and mitochondria transplantation [40]. Further studies are still needed to confirm whether preoperative metabolic intervention will improve surgical outcomes in aging patients.

## Funding

This work was supported by National Key R&D Program of China ) (grant number 2018YFC2001800); 10.13039/501100001809National Natural Science Foundation of China (grant number 82001183); CAMS Innovation Fund for Medical Sciences (grant number 2019-I2M-5–011); 135 project for disciplines of excellence, 10.13039/501100013365West China Hospital, Sichuan University (grant number ZYJC21008); National Postdoctoral Researcher Program (GZC20231834).

## Data availability

The raw data supporting the conclusions of this article was included in article and will be made available by contacting the authors, without undue reservation.

## Ethics declarations

Ethic approval for this study has been obtained from the Ethics Committee on Biomedical Research, West China Hospital of Sichuan University, Sichuan, China (No. 2019-624). This trial was registered at Chinese Clinical Trial Registry (ChiCTR1900026223). All participants or their legal guardians provided informed consent to participate in the study.

## CRediT authorship contribution statement

**Yi Zhao:** Writing – review & editing, Writing – original draft, Methodology, Investigation. **Mengchan Ou:** Methodology, Investigation, Funding acquisition. **Xuechao Hao:** Funding acquisition, Formal analysis, Conceptualization. **Tao Zhu:** Resources, Funding acquisition, Conceptualization.

## Declaration of competing interest

The authors declare that they have no known competing financial interests or personal relationships that could have appeared to influence the work reported in this paper.
