# Supplementary material for: Metabolic change in monocytes and postoperative morbidity after major abdominal surgery in elderly patients: A prospective cohort study
Source: Heliyon. 2024 Mar 22;10(7):e28137. doi: 10.1016/j.heliyon.2024.e28137 (PMC10987940; doi:10.1016/j.heliyon.2024.e28137)
Supplement: Multimedia component 1 [file mmc1.docx]

STROBE Statement—Checklist of items that should be included in reports of ***cohort studies***

|  | Item No | Recommendation | Page No |
| --- | --- | --- | --- |
| **Title and abstract** | 1 | (*a*) Indicate the study’s design with a commonly used term in the title or the abstract | Page1, line 2 |
|  |  | (*b*) Provide in the abstract an informative and balanced summary of what was done and what was found | Page4-5, line 57-83 |
| Introduction | | | |
| Background/rationale | 2 | Explain the scientific background and rationale for the investigation being reported | Page6-8, line 67-109 |
| Objectives | 3 | State specific objectives, including any prespecified hypotheses | Page6, line 107-159 |
| Methods | | | |
| Study design | 4 | Present key elements of study design early in the paper | Page8, line 163 |
| Setting | 5 | Describe the setting, locations, and relevant dates, including periods of recruitment, exposure, follow-up, and data collection | Page8, line 163-179 |
| Participants | 6 | (*a*) Give the eligibility criteria, and the sources and methods of selection of participants. Describe methods of follow-up | Page 8, line 169-179  Page 11, line 238-243 |
|  |  | (*b*) For matched studies, give matching criteria and number of exposed and unexposed | Not relevant |
| Variables | 7 | Clearly define all outcomes, exposures, predictors, potential confounders, and effect modifiers. Give diagnostic criteria, if applicable | Page11. Lin240-243 |
| Data sources/ measurement | 8* | For each variable of interest, give sources of data and details of methods of assessment (measurement). Describe comparability of assessment methods if there is more than one group | Page8-10, line 180-217 |
| Bias | 9 | Describe any efforts to address potential sources of bias | Page8, line178-179 |
| Study size | 10 | Explain how the study size was arrived at | Page12, line 258-265 |
| Quantitative variables | 11 | Explain how quantitative variables were handled in the analyses. If applicable, describe which groupings were chosen and why | Page11-12, line 245-257 |
| Statistical methods | 12 | (*a*) Describe all statistical methods, including those used to control for confounding | Page11-12, line 245-257 |
|  |  | (*b*) Describe any methods used to examine subgroups and interactions | Not relevant |
|  |  | (*c*) Explain how missing data were addressed | Page12, line 264-265 |
|  |  | (*d*) If applicable, explain how loss to follow-up was addressed | Page12, line 264-265 |
|  |  | (*e*) Describe any sensitivity analyses | Not relevant |
| Results | | |  |
| Participants | 13* | (a) Report numbers of individuals at each stage of study—eg numbers potentially eligible, examined for eligibility, confirmed eligible, included in the study, completing follow-up, and analysed | Page 12, line 268-273 |
|  |  | (b) Give reasons for non-participation at each stage | Page 12, line 269-271 and Figure 2 |
|  |  | (c) Consider use of a flow diagram | Figure 2 |
| Descriptive data | 14* | (a) Give characteristics of study participants (eg demographic, clinical, social) and information on exposures and potential confounders | Page12, line 273-275 and Table 1 |
|  |  | (b) Indicate number of participants with missing data for each variable of interest | Page12, line 271-273 |
|  |  | (c) Summarise follow-up time (eg, average and total amount) | Not relevant |
| Outcome data | 15* | Report numbers of outcome events or summary measures over time | Page13, line 284-286 |

| Main results | 16 | (*a*) Give unadjusted estimates and, if applicable, confounder-adjusted estimates and their precision (eg, 95% confidence interval). Make clear which confounders were adjusted for and why they were included | Page 12-13,line 219-259 |
| --- | --- | --- | --- |
|  |  | (*b*) Report category boundaries when continuous variables were categorized | Not relevant |
|  |  | (*c*) If relevant, consider translating estimates of relative risk into absolute risk for a meaningful time period | Not relevant |
| Other analyses | 17 | Report other analyses done—eg analyses of subgroups and interactions, and sensitivity analyses | Not relevant |
| Discussion | | | |
| Key results | 18 | Summarise key results with reference to study objectives | Page13, line 262-273 |
| Limitations | 19 | Discuss limitations of the study, taking into account sources of potential bias or imprecision. Discuss both direction and magnitude of any potential bias | Page15-16, line 325-331 |
| Interpretation | 20 | Give a cautious overall interpretation of results considering objectives, limitations, multiplicity of analyses, results from similar studies, and other relevant evidence | Page 16, line 332-344 |
| Generalisability | 21 | Discuss the generalisability (external validity) of the study results | Page 16, line 340-344 |
| Other information | | | |
| Funding | 22 | Give the source of funding and the role of the funders for the present study and, if applicable, for the original study on which the present article is based | Page 117, line 352-357 |

*Give information separately for exposed and unexposed groups.

**Note:** An Explanation and Elaboration article discusses each checklist item and gives methodological background and published examples of transparent reporting. The STROBE checklist is best used in conjunction with this article (freely available on the Web sites of PLoS Medicine at http://www.plosmedicine.org/, Annals of Internal Medicine at http://www.annals.org/, and Epidemiology at http://www.epidem.com/). Information on the STROBE Initiative is available at http://www.strobe-statement.org.
