# Supplementary material for: Metabolic change in monocytes and postoperative morbidity after major abdominal surgery in elderly patients: A prospective cohort study
Source: Heliyon. 2024 Mar 22;10(7):e28137. doi: 10.1016/j.heliyon.2024.e28137 (PMC10987940; doi:10.1016/j.heliyon.2024.e28137)
Supplement: Multimedia component 2 [file mmc2.docx]

**Supplemental method**

**Definition of preoperative comorbidities**

Hypertension was defined if the patient had previous medical documentation of hypertension and is currently taking antihypertensive medication. Coronary artery disease is defined as a history of angina; myocardial infarction; positive exercise, a nuclear or echocardiographic stress test; resting wall motion abnormalities on echocardiogram; coronary angiography with evidence of ≥ 50% vessel stenosis; or an electrocardiogram with pathologic Q-waves in 2 contiguous leads. A requirement for insulin or oral hypoglycemic therapy at the time of admission for surgery was considered to be diabetes mellitus. Arrhythmias were considered to be any type of previously diagnosed arrhythmias or abnormal ECG after admission. A patient will be considered a smoker, if they had a history of smoking within 1 year before surgery. Chronic obstructive pulmonary disease (COPD) was based on past medical history. Cerebrovascular disease was defined as a previous cerebrovascular accident or transient ischemic attack.

**Supplemental Table 1. Metabolic changes during perioperative period.**

| **Variables** | **Value, median [IQR]** | | **P value** |
| --- | --- | --- | --- |
|  | **Preoperative** | **Postoperative** |  |
| **Mitochondrial respiration** | | | |
| BR | 205.80 [158.60, 251.33] | 210.70 [161.10, 272.40] | 0.188 |
| PL | 24.05 [15.30, 33.45] | 24.35 [12.83, 34.90] | 0.935 |
| MR | 457.40 [321.33, 636.60] | 488.90 [342.58, 642.78] | 0.325 |
| SRC | 249.14 [136.98, 376.85] | 280.55 [158.02, 387.07] | 0.104 |
| NMR | 76.50 [58.80, 94.73] | 79.95 [60.73, 93.80] | 0.447 |
| ATP-P | 176.20 [135.92, 221.23] | 189.25 [140.80, 234.53] | 0.081 |
| BHI | 1.39 [1.11, 1.60] | 1.49 [1.14, 1.69] | 0.008 |
| **Glycolytic function** | | | |
| NG | 5.74 [4.26, 7.62] | 7.91 [5.40, 10.50] | <0.001 |
| G | 1.17 [0.61, 2.01] | 1.78 [1.05, 3.05] | <0.001 |
| GC | 5.38 [3.51, 8.00] | 6.00 [4.00, 9.20] | 0.062 |
| GR | 3.77 [2.21, 6.56] | 3.70 [3.39, 6.66] | 0.850 |
| GR/GC | 0.75 [0.61, 0.90] | 0.66 [0.54, 0.79] | <0.001 |
| GR/G | 2.94 [1.50, 8.54] | 1.92 [1.16, 3.77] | 0.001 |
| GR/NG | 0.69 [0.41, 0.10] | 0.51 [0.33, 0.81] | 0.002 |

IQR, inter-quartile range; OCR, oxygen consumption rate; ECAR, extracellular acidification rate; BR, basal respiration; PL, proton leak; MR, maximal respiration; SRC, spare respiration capacity; NMR, non-mitochondrial respiration; ATP-P, ATP production; BHI, bioenergetic health index; NG, non-glycolytic acidification; G, glycolysis; GC, glycolytic capacity; GR, Glycolytic Reserve.

**Supplemental Table 2. Comparison paraments between complication positive and complication negative patients.**

| **Variables** | **OCR group (n=110),** **median [IQR]** | | | **Variables** | **ECAR group (n=122), median [IQR]** | | |
| --- | --- | --- | --- | --- | --- | --- | --- |
|  | **≥ CDC grade 3 (n=14)** | **< CDC grade 3 (n=96)** | ***P* value** |  | **≥ CDC grade 3 (n=15)** | **< CDC grade 3 (n= 107)** | ***P* value** |
| Preoperative | |  |  |  |  |  |  |
| BR | 206.65 [169.73, 264.35] | 205.81 [154.15, 249.52] | 0.470 | NG | 6.03 [4.51, 10.41] | 5.69 [4.14, 7.55] | 0.332 |
| PL | 25.05 [19.95, 30.93] | 23.45 [15.30, 34.82] | 0.994 | G | 1.40 [0.59, 2.09] | 1.14 [0.61, 2.04] | 0.3803 |
| MR | 466.15 [377.78, 617.73] | 442.23 [316.53, 641.84] | 0.496 | GC | 5.72 [3.82, 9.87] | 5.29 [3.41, 7.91] | 0.436 |
| SRC | 253.24 [188.83, 390.55] | 246.58 [123.73,378.15] | 0.447 | GR | 4.78 [2.39, 7.81] | 3.71 [2.05, 6.48] | 0.362 |
| NMR | 77.83 [60.13, 88.12] | 76.35 [58.83, 95.13] | 0.889 | GR/GC | 0.85 [0.64, 0.92] | 0.74 [0.60, 0.89] | 0.163 |
| ATP-P | 181.15 [150.65, 236.83] | 176.22 [132.03, 219.23] | 0.441 | GR/G | 5.73 [1.75, 12.00] | 2.80 [1.48, 8.50] | 0.140 |
| BHI | 1.46 [1.25, 1.60] | 1.39 [1.04, 1.60] | 0.534 | GR/NG | 0.76 [0.44, 1.04] | 0.67 [0.38, 0.99] | 0.726 |
| Postoperative | |  |  |  |  |  |  |
| BR | 189.35 [164.00, 237.73] | 212.58 [1.53, 273.43] | 0.633 | NG | 10.19 [5.47, 12.23] | 7.89 [5.34, 10.41] | 0.328 |
| PL | 15.95 [6.16, 33.86] | 24.54 [13.28, 35.11] | 0.192 | G | 1.23 [1.03, 2.91] | 1.87 [1.11, 3.11] | 0.308 |
| MR | 488.93 [3.75, 548.88] | 487.05 [341.73, 681.57] | 0.739 | GC | 3.89 [3.01, 9.11] | 6.13 [4.27, 9.46] | 0.140 |
| SRC | 306.65 [169.14, 364.03] | 274.55 [157,48, 409.39] | 0.891 | GR | 3.18 [1.83, 6.62] | 3.72 [2.44, 6.84] | 0.409 |
| NMR | 69.72 [57.35, 90.33] | 81.95 [61.24, 94.03] | 0.280 | GR/GC | 0.71 [0.50, 0.76] | 0.65 [0.54, 0.79] | 0.897 |
| ATP-P | 166.31 [1.40, 218.74] | 192.15 [139.88, 237.94] | 0.687 | GR/G | 2.43 [1.00, 3.18] | 1.88 [1.16, 3.83] | 0.848 |
| BHI | 1.67 [1.43, 1.80] | 1.48 [1.13, 1.66] | 0.053 | GR/NG | 0.35 [0.15, 0.57] | 0.53 [0.34, 0.81] | 0.047 |
| Preoperative - postoperative | |  |  |  |  |  |  |
| BR | 14.75 [-54.38, 73.59] | -14.1 [-85.04, 52.75] | 0.437 | NG | -1.35 [-6.14, 1.31] | -1.64 [-4.36, 0.34] | 0.803 |
| PL | 5.73 [-4.43, 16.43] | 0.11 [-14.76, 12.41] | 0.284 | G | -0.62 [-1.40, 0.63] | -0.53 [-2.13, 0.21] | 0.706 |
| MR | 53.85 [-171.88, 177.67] | -58.25 [-245.13, 137.03] | 0.437 | GC | 0.76 [-1.96, 3.18] | -0.98 [-4.02, 1.68] | 0.202 |
| SRC | 32.15 [-170.93, 166.84] | -0.48.4 [-188.58, 95.11 | 0.317 | GR | 1.28 [-0.98, 1.87] | -0.23 [-0.23, 2.21] | 0.194 |
| NMR | 1.62 [-12.58, 21.96] | -8.00 [-26.14, 23.45] | 0.481 | GR/GC | 0.12 [0.05, 0.25] | 0.04 [-0.08, 0.21] | 0.114 |
| ATP-P | 16.34 [-59.63, 55.48] | -15.45 [-85.65, 42.52] | 0.358 | GR/G | 3.01 [0.57, 8.73] | 0.52 [-1.11, 2.99] | 0.018 |
| BHI | -0.27 [-0.48, 0.07] | -0.10 [-0.44, 0.20] | 0.229 | GR/NG | 0.32 [0.12, 0.63] | 0.30 [-0.15, 0.47] | 0.029 |

IQR, inter-quartile range; OCR, oxygen consumption rate; ECAR, extracellular acidification rate; CDC, Clavien-Dindo Classification; BR, basal respiration; P, proton leak; MR, maximal respiration; SRC, spare respiration capacity; NMR, non-mitochondrial respiration; ATP-P, ATP production; BHI, bioenergetic health index, NG, non-glycolytic acidification; G, glycolysis; GC, glycolytic capacity; GR, Glycolytic Reserve.
